# The influence of ecological and geographical context in the radiation of Neotropical sigmodontine rodents

**DOI:** 10.1186/s12862-015-0440-z

**Published:** 2015-08-26

**Authors:** Andrés Parada, Guillermo D’Elía, R. Eduardo Palma

**Affiliations:** 1 Instituto de Ciencias Ambientales y Evolutivas, Facultad de Ciencias, Universidad Austral de Chile, Valdivia, Chile; 2 Departamento de Ecología, Laboratorio de Biología Evolutiva, Pontificia Universidad Católica de Chile, Santiago, Chile

## Abstract

**Background:**

Much debate has focused on how transitions in life history have influenced the proliferation of some clades. Rodents of the subfamily Sigmodontinae (family Cricetidae) comprise one of the most diverse clades of Neotropical mammals (~400 living species in 86 genera). These rodents occupy a wide range of habitats and lifestyles so that ecological context seems relevant to understand the evolution of this group. Several changes in the landscape of South America through the Neogene might have provided vast resources and opportunity to diversify. The aim of this study was to examine whether transitions between i) lowland and montane habitats, ii) open vegetation and forest, and iii) distinct molar architectures are correlated with shifts in diversification rates and to characterize the general pattern of diversification.

**Results:**

Based on a dense taxon sampling of 269 species, we recovered a new phylogeny of Sigmodontinae that is topologically consistent with those of previous studies. It indicates that the subfamily and its major lineages appeared during the Late Miocene. Analyses suggest that vegetation type and elevational range are correlated with diversification rates, but not molar architecture. Tropical lowlands accumulated more lineage diversity than other areas and also supported high speciation rates. Across the radiation the subfamily Sigmodontinae appear to have experienced a decline in diversification rate through time. We detected mixed evidence for lineage-specific diversification rate shifts (e.g., leading to the clades of *Akodon, Bibimys, Calomys* and *Thomasomys*).

**Conclusion:**

We report that the evolution of habitat preference (considering vegetation type and elevational range) was associated with diversification rates among sigmodontine rodents. We propose that the observed diversification slowdown might be the result of ecological or geographical constraints. Our results also highlight the influence of the tropical lowlands -which might have acted as both “a cradle and a museum of species.” The tropical lowlands accumulated greater diversity than the remainder of the group's range.

**Electronic supplementary material:**

The online version of this article (doi:10.1186/s12862-015-0440-z) contains supplementary material, which is available to authorized users.

## Background

The uneven distribution of species richness and why some clades fail to radiate while others proliferate are among the most impressive features of biological diversity. Nowadays, comparative methods facilitate inferring the tempo and mode of evolution at macroevolutionary scales (e.g., [1, 2]). The rich biota of South America [3–6] with its extended period of isolation and numerous episodes of faunal interchanges [7] provides multiple scenarios to examine broad-scale patterns of diversity.

The subfamily Sigmodontinae, with about 400 living species and 86 living genera, is one of the most species-rich and broadly distributed groups of Neotropical mammals [6, 8]. Sigmodontine genera have been traditionally assembled into groups, most of which are formally recognized tribes [9–11] that greatly differ in specific and ecological diversity [9]. For example, Reithrodontini has one living genus with two herbivorous species that inhabit open areas of southern South America; whereas the tribe Oryzomyini encompasses about 34 genera and 130 living species of typical mice and rats, of mostly cursorial and some arboreal or semiaquatic species that inhabitforested areas [8]. Changes in the landscape of South America during the course of the Neogene and colonization of new areas might have provided ample ecological opportunity [12, 13] -i.e. “a wealth of evolutionarily accessible resources little used by competing taxa” [14]- and opportunity to diversify. The coupling of tectonic and climatic processes resulted in changes of the Andean flanks and eastern lowlands leading to the emergence of more “biotic corridors” and new habitats (e.g., [15, 16]). The Andean highlands may have promoted *in situ* species accumulation [9] and then some offshoots of this initial radiation may have diversified through recently colonized lowlands. Vegetational changes in the Late Miocene triggered the advance of savannas and more open vegetation habitats [17] providing more opportunities to proliferate. Furthermore, transitions in molar architecture might have enabled some lineages to exploit or gain access to a novel variety of food resources. It has been proposed that the appearance of a molar with four lophs, known as tetralophodont (usually high crowned or hypsodont), facilitated the access to grazing pastures and allowed the colonization of open or “pastoral” habitats, while the plesiomorphic type of five lophs, or pentalophodont plan (usually low crowned or brachydont), remained associated with taxa from forested habitats [18].

In addition to Reig and Hershkovitz [9, 18, 19], other researchers have evaluated the timing of diversification (e.g., [20, 21]) and other aspects of the sigmodontine radiation. For instance, analyses of habitat preference inferred 4 transitions between open and forested habitats for the tribe Oryzomyini ([22, 23]). Regarding the tempo of diversification, studies have found quick burst of diversification at the base of the speciose clade of Oryzomyalia [24–26]. Therefore, it would be of much interest to test whether there are differences in speciation rates among lineages inhabiting distinct habitat types. Similarly, it is of interest to assess if molar types are associated with differential diversification rates.

Here we constructed the most complete and dated phylogeny of Sigmodontinae and allies based on sequences of a combined mitochondrial and nuclear gene matrix for 290 species and three internal fossil-based constraints. Our goals were to: i) reconstruct the evolution of main habitat type preferences (i.e., elevational range and occurrence in open or forested habitats) and molar morphology; ii) test whether among-lineage variation in diversification rates explain the observed disparity among clades; iii) test whether transitions in habitat type preferences (elevation, vegetation) and/or molar architecture have led to changes in diversification rates; and iv) examine whether the Andean or Amazonian regions sustained greater diversification rates than other areas.

## Methods

### Taxon and gene sampling

Analyses were based on DNA sequences of the first exon of the nuclear gene interphotoreceptor retinoid binding protein (IRBP) and the mitochondrial cytochrome b (cyt b); all sequences were gathered from GenBank (Additional file 1: Table S1). A total of 290 species of superfamily Muroidea were recovered. We included 269 sigmodontine species, including representatives of all recognized tribes as well as several species considered *incertae sedis* [8, 11]. For those taxa which either no mitochondrial (6 cases) or nuclear genes (120 cases) were available, the matrix was completed with missing state characters (i.e., n). To infer the placement of Sigmodontinae relative to other rodents, our coverage considered 21 muroid species as the outgroup. We included sequences from two representatives of each of the other cricetid subfamilies and one representative of each non-cricetid subfamilies of Muroidea (following [24, 26]; our sample lacks representative of Leimacomyinae). Notwithstanding, our taxonomic sampling is so far the most extensive of any study focusing on sigmodontine evolutionary history.

### Phylogenetic reconstruction and divergence time estimates

Sequences were aligned with MAFFT v.6.925b [27] with auto settings. The IRBP matrix had 1181 positions of which 751 were variable. The cyt b matrix had 1134 positions and 874 variable sites. A Bayesian analysis in BEAST v1.7.4 [28] simultaneously estimated the topology, substitution model parameters and dates for cladogenetic events. A Birth-Death [BD] process with incomplete sampling [29] using an initial random tree and other priors set as default were used. The employed substitution models, selected using ModelGenerator [30], were TVM (transversional model) + γ + I for the cyt b gene and TVM + γ for the IRBP gene (implemented by modifying the file generated with the program BEAUti), with empirical base frequencies, and four γ rate categories. Runs were performed under an uncorrelated lognormal relaxed-clock model. Three independent runs of 9.8 × 10^7^ generations, sampled every 20 × 10^3^ generations were performed. Convergence to stable values was checked with Tracer v.1.6 [31] obtaining an effective sample size (ESS) greater than 200 for all parameters. Tree and log files (14325 trees after a 2.55 % burn-in) were combined using LogCombiner [28]. Trees then were compiled into a maximum clade credibility (MCC) tree using TreeAnnotator [28] to display mean node ages and highest posterior density (HPD) intervals (95 % upper and lower) for each node. Fossil calibrations were employed as lognormal prior distributions, providing a minimum bound for each distribution such that the 5 % quantile corresponds to the minimum age of the fossil while the 95 % interval accounts both for the uncertainty of the fossil age and for the incompleteness of the fossil record. We used the following calibrations [offset, median, 5 % and 95 % quantiles in million year ago (Mya) respectively]: (a) the crown clade aff. *Abrothrix* (1.99, 3.00, 2.61 and 3.64) based on the fossil aff. *Abrothrix* [32], (b) the crown clade of *Sigmodon* (4.13, 5.14, 4.506 and 6.84) based on the fossil record of *Sigmodon* spp. [33], and (c) the crown clade of Phyllotini (4.95, 5.96, 5.326 and 7.66) based on the fossil species *Auliscomys formosus* Reig [9, 34].

### Diversification analyses using γ-statistic and a model-fitting approach.

To examine the tempo and mode of diversification of Sigmodontinae we used R version 3.0.1 [35] in combination with the packages ape [36], diversitree [37], GEIGER [38], LASER [39], phytools [40], TreePar [41] and TreeSim [42].To correct for any sampling bias in our data, we applied an objective automated method for fitting diversification models to non-randomly sampled phylogenies. The CorSiM approach [43] simulated missing splits under a constant rate birth–death model and took into account that species sampling in our phylogeny was nonrandom using TreePar and TreeSim. Missing branching times were simulated -under a constant birth-death model- and then added to the empirical branching times, yielding 1000 “*completed*” data sets (scripts available at [44]).

To visualize the pattern of diversification we constructed lineage-through-time (LTT) plots for the MCC tree and the *completed* and data sets. We also simulated 1000 trees under a pure birth [PB] model -using *pbtree* in phytools- and plotted the mean LTT of these simulated trees and a 95 % CI. To test for constant rates of diversification we calculated the gamma-statistic for the MCC tree and *completed* trees and we applied the MCCR test with 4000 replicas [45]. We then applied a model-fitting strategy in Laser and TreePar for the MCC tree and the *completed* data sets. We estimated the overall diversification rate under a simple BD model, a PB model, and PB and BD models extended to have a varying speciation rate (λ) sin 2 intervals (Y2r and B2r respectively, [1]). We also tested two models that predict slowdowns in the tempo of diversification (density-dependent exponential and density-dependent logistic (DDX and DDL, [46]). In order to examine if the pattern of diversification is the result of past slowdowns in speciation rate, increased extinction rates, or both, we considered the SPVAR, EXVAR, and BOTHVAR models. We used Akaike’s Information Criterion (AIC) scores to compare the fit of these models.

### Macroevolutionary dynamics and rate shifts

We tested for differential diversification rates with two strategies. Firstly, we used MEDUSA [2] to fit both PB and BD models to estimate rate shifts on a dated phylogeny accommodating incomplete sampling by using taxonomic richness information. To do so, the MCC tree was pared down to a backbone tree, with sigmodontine species (n = 365) assigned, following [8], to the 79 tips representing distinct genera with the exception of seven tips (Additional file 2: Table S2). Thirty-five species were excluded from these analyses because they do not belong to the 79 genera examined in the matrix. The first MEDUSA analysis was performed with the backbone tree. To account for topological uncertainty of our reconstruction we randomly selected from the posterior distribution of the analysis done with BEAST a subset of 6000 trees and reran MEDUSA with these trees. Secondly, we employed BAMM, a method recently developed to detect and quantify heterogeneity in evolutionary rate [47, 48], which differs from other methods because it does not assume constant evolutionary rates through time. We conducted 120 × 10^6^ generations of MCMC, sampling each 24 × 10^3^ generations, using four Markov chains, and a “minimum clade size for shift” of two. To account for our non-random incomplete sampling, we provided the relevant sampling fractions for each lineage in the MCC tree (as described at [49]). We assessed convergence of BAMM runs by computing ESS for the likelihood of the data and for the number of distinct regimes, obtaining more than 4000 independent samples from the posterior after a “burn-in” of 10 %. We plotted a rate-through-time curve to examine whether there is variation in the speciation and extinction rates. We identified a credible set of distinct shift configurations (distinguished by the presence or absence of shifts) that account for 95 % of the probability of the data. These “cores shifts” were determined based on a Bayes factor (BF) above 5. To measure the evidence supporting rate shifts, the BF associated with rate shift on particular branches of our phylogeny were computed. Macroevolutionary dynamics across any two lineages were illustrated by macroevolutionary cohort analysis [50].

### Ancestral state Reconstruction and correlates of diversification

We examined three traits hypothesized to be correlated with differential rates of diversification in Sigmodontinae: (i) elevational range, (ii) habitat or vegetation type, and (iii) molar plan. We categorized elevational range and habitat in the sampled taxa as characters with three broad states. First, species were coded according to their elevational range as: a) lowlands (from 0 to 800 m); b) middle range (up to 3000 m); or c) highland (above 3000 m). Second, species were coded as: a) occurring in open vegetation habitats such as grasslands and scrublands; b) forest; or c) occurring in both habitats, fringe habitats or a mixture of both type of habitats (hereafter “mixed”). Molar type was coded as a binary character: a) tetralophodont; or b) pentalophodont. Elevational range and habitat type data were obtained from the catalog of Musser and Carleton [51, 52] updated with information displayed at the IUCN site [52] and primary literature [see [8] and references therein] . Molar plan was compiled primarily after [53] and [22]. Ancestral state reconstruction considering the MCC tree was performed in phytools, which fits a continuous-time reversible Markov model for trait evolution and then simulates stochastic character histories. For this procedure, taxa with missing or ambiguous information for these traits (4, 9 and 6 taxa for altitude, vegetation type and molar type respectively) were excluded from the analysis.

If entering into novel habitat types gave sigmodontine rodents an opportunity for cladogenesis, diversification rates would be higher for lineages in these habitats. We used the MuSSE (Multiple State Speciation Extinction) model -implemented in diversitree [54] - to test whether lineage-specific diversification rates differed across distinct elevational range or vegetation type preferences. We compared with AIC the fit of a model where the speciation rate was independent of habitat to models where distinct speciation rates changed along the altitudinal range. We calculated parameters for the unconstrained full model and then sequentially constrained each of the model parameters, alone and in combination. This protocol allowed contrasting a simple model with a constant rate during the radiation of the group with more complex models that incorporate rate-variation across lineages. We used a sampling fraction of 0.6725 to account for the incomplete taxon sampling of our MCC tree. We constrained the full model into three alternative models and one model allowing parameters to vary according to two a posteriori established “epochs” established at one point in the past that is inferred via likelihood in diversitree. We followed a similar procedure to test whether preference for distinct vegetation types were associated with differential rates. Finally, we tested whether transition in molar plan correlates with differential rates of diversification using the BISSE (Binary State Speciation and Extinction) model [55], also implemented in diversitree. Bayesian posterior distributions of model parameters were estimated in diversitree using Markov Chain Monte Carlo analyses and 8000 generations per chain for the aforementioned traits. All these analyses were performed considering the MCC tree.

### Geographic range evolution

The geographic range for each species was obtained from the catalog of Musser and Carleton [56], updated with ranges portrayed in IUCN [52] and recent literature [8]. Biogeographic regions corresponded to those regions, subregions or provinces defined by Morrone [57]. We tested if transitions into new geographic areas provided Sigmodontinae opportunity to diversify; specifically we tested if inhabiting the Andes or tropical lowlands is correlated with higher speciation rates. In order to consider simple models we established two classification schemes. For the first model (hereafter “Andean model”) species distribution was set as a) present in, or b) absent from an area composed of Andean, Paramo, Patagonian, and Puna regions. For the second model (hereafter “tropical lowland model”), we treated the distribution as either a) present or b) absent of an area composed by the tropical lowlands of Amazonia, Parana, Cerrado, Caatinga and also the Yungas. Since the Yungas is a tropical forest-highland transitional zone, we also rerun these models excluding this province from the “tropical lowland” distribution and including it as part of the “Andean” distribution. We ran these analyses with the ClaSSE (Cladogenetic State change Speciation and Extinction) model [58] function in the diversitree package. This likelihood-based approach allowed the estimation of region-dependent rates of speciation and extinction as well as range evolution. To reduce the complexity of the analysis, 8 ClaSSE models (i.e., a full-parameter model and others with fewer parameters) were evaluated under a ML framework and compared using AIC. As in previous trait analyses, we used a sampling fraction of 0.6725 to account for the missing taxa. Finally, employing phytools, we estimated the lineage density at each node considering the geographic distribution with a method based on Mahler et al. [59] with the function estDiversity under the “simulation” method [40]. The accumulation of lineages in each region is illustrated as nodal “lineage density” that is “the number of co-existing lineages with the same biogeography as the focal node, averaged across stochastic maps” (see also phytools manual [40]). This visualization of lineage accumulation includes in situ diversification and diversity arising from immigration into a particular area (see [60]). All these analyses were performed considering the MCC tree.

## Results

### Phylogenetic analysis

**Fig. 1:**
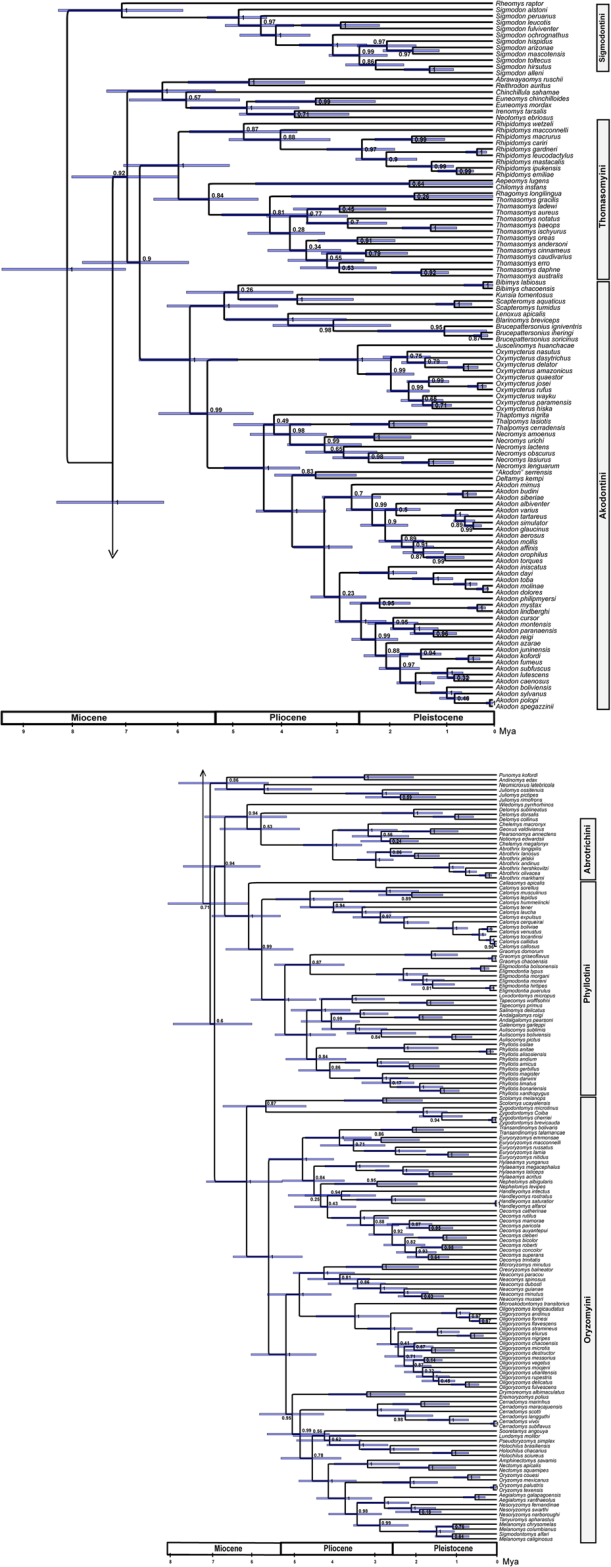
Maximum clade credibility tree for the subfamily Sigmodontinae obtained with BEAST using 290 species. Calibration points considered in the analysis are highlighted with circles. Numbers on nodes indicate posterior probability for the corresponding nodes. Bars represent the 95 % highest posterior density (HPD) interval for the divergence times. Tribes indicated on the right.

Chronograms were compiled and annotated from 14325 post-burnin trees as a MCC tree (Highest Log Clade Credibility: −45.856), whose topology was highly congruent to those gathered in previous studies (e.g., [10, 20–26, 61, 62]). In particular, as in previous studies (see Table 1), the monophyly of the subfamily Sigmodontinae (PP = 1; Fig. 1) as well as the main sigmodontine lineages were recovered. The most basal split within Sigmodontinae led to one clade (PP = 1, composed of *Sigmodon* (PP = 1) and *Rheomys*, and to another clade (PP = 1), named by Steppan et al. [24] Oryzomyalia, which contains the remaining sigmodontines. Within the later, monophyly was strongly supported (all with PP = 1) for all tribes (Abrotrichini, Akodontini, Oryzomyini, Phyllotini,Thomasomyini) containing one or more representative genera. In addition, a clade composed of the *insertae sedis Abrawayaomys*, *Chinchillula*, *Euneomys*, *Irenomys*, *Neotomys*, and *Reithrodon* (PP = 1) was recovered. As in earlier studies (e.g., [10, 20–26, 61, 62], relationships among tribes of Oryzomyalia lacked significant support.

**Table 1:** Comparison of the phylogenetic results obtained in the present study and those of selected previous phylogenies of Sigmodontinae. Authors, loci analyzed, number of sigmodontine species/genera included, method of reconstruction (B: Bayesian; ML: Maximum Likelihood; MP: Maximum parsimony), and measure of nodal support employed (BS: Bootstrap support;, JK: Jackknife; PP: Posterior Probability) are given for each publication. Support values are given for the main clades of the sigmodontine radiation

Authors	Smith and Patton 1999	Steppan et al. 2004	D’Elía et al. 2006	Fabre et al. 2012	Parada et al. 2013	Schenk et al. 2013	Leite et al. 2014	This study
Loci	cytb	c-myc/ BRCA1/GHR /RAG1/	IRBP	12S rRNA/BRCA1/CR/COX3/cytb /IRBP/GHR/NAD H1/NADH4/RAG1 /vWF	cytb/IRBP	BRCA1/ GHR/IRBP/RAG1	cytb/IRBP	cytb/IRBP
Number spp/genera	85/38	10/10/15	39/39	231/72	72/72	76/50	66/54	269/76
Reconstruction Method	MP	ML/B	MP	ML	B	B/ML	B	B
Support Measure	BS	BS	J	BS	PP	PP/BS	PP	PP
Clade								
Sigmodontinae	93	100/1	100	99	1	*/100	1	1
Sigmodontalia		---	100	70-95	0.9	*/100	1	1
Sigmodontini		---		>95	1	*/100	1	1
Oryzomyalia		100/1	100		0.99	*/100	1	1
Abrotrichini	79	---	100	>95	1	*/100	0.94	1
Sigmodontinae	93	100/1	100	99	1	*/100	1	1
Sigmodontini				>95	1	*/100	1	1
Sigmodontalia			100	70-95	0.9	*/100	1	1
Akodontini		---	99	70-95	1	*/100	1	1
Oryzomyini		---	100	70-95	1	*/100	1	1
Phyllotini		100	89	>95	1	*/99	1	1
Thomasomyini		---	72	50-70	0.92	*/99	0.81	1
Phyllotini		100	89	>95	1	*/99	1	1
Oryzomyini			100	70-95	1	*/100	1	1
Oryzomyalia		100/1	100		0.99	*/100	1	1

*Abbreviations:* *BRCA1* = Breast and ovarian cancer susceptibility protein exon 11, *c-myc* = C-myc intron 2, *CR* = control region, *cytb* = cytochrome b, *COX3*= cytochrome oxidase 3; *GHR* = growth hormone receptor, *IRBP* = Interphotoreceptor retinol-binding protein exon 1, *NADH1* = NADHdehydrogenase 1, *NADH4* = NADH dehydrogenase 4, *RAG1* = recombination activating protein exon 1, *vWF* = von Willebrand gene exon 28

* PP value between 0.95-1. --- indicates that the taxonomic sampling made not posible to test the monophyly of the given group

### Divergence time estimates

Our relaxed clock inferred a stem age of 9.91 Mya (95 % HPD 8.42-11.53) for Sigmodontinae; meanwhile the crown age of Sigmodontinae was inferred as 8.12 Mya (95 % HPD 7.01-9.36). During the Late Miocene and Pliocene, several splits lead to the emergence of most of the sigmodontine tribes (Abrotrichini, Akodontini, Oryzomyini, Phyllotini, Reithrodontini, Thomasomyini, and Wiedomyini). Throughout the Late Miocene the crown groups of the tribes Akodontini, Oryzomyini, Phyllotini, and Thomasomyini began to diversify (see Table 2). The stem lineages of Abrotrichini and Sigmodontini began to radiate subsequently, in the Pliocene. The stem lineages leading to the clades containing *Abrawayaomys* and *Punomys* appeared close to the base of the radiation during Late Miocene or Early Pliocene.

**Table 2:** Comparison of time estimates for the sigmodontine radiation obtained in the present and previous studies. Age estimate and 95 % highest posterior probability (HPD) in millions of years are shown. Authors of the study are indicated in the first row

Clade	Parada et al. 2013		Leite et al. 2013		This study	
	Age	95 % HPD	Age	95 % HPD	Age	95 % HPD
Abrotrichini	4.92	3.93–6.02	3.9	3.5−4.4	4.05	3.33−4.77
Akodontini	7.36	5.62−9.29	6.1	5.1−7.2	5.78	4.86−6.75
Oryzomyalia	9.81	7.68−12.08	8.2	7.3−9.2	7.25	6.28−8.32
Oryzomyini	7.72	6.01−9.64	6.6	5.7−7.5	6.21	5.29−7.16
Phyllotini	6.93	5.45−8.54	5.3	4.9−6.0	6.12	5.33−7.03
Sigmodontini	5.08	4.33−6.02			4.85	4.31−5.45
Sigmodontinae	11.82	9.28−14.70	9.6	8.5−10.8	8.12	7.01−9.36
Thomasomyini	8.24	6.11−10.66			6	5.02−7.04

### Diversification analyses

**Fig. 2:**
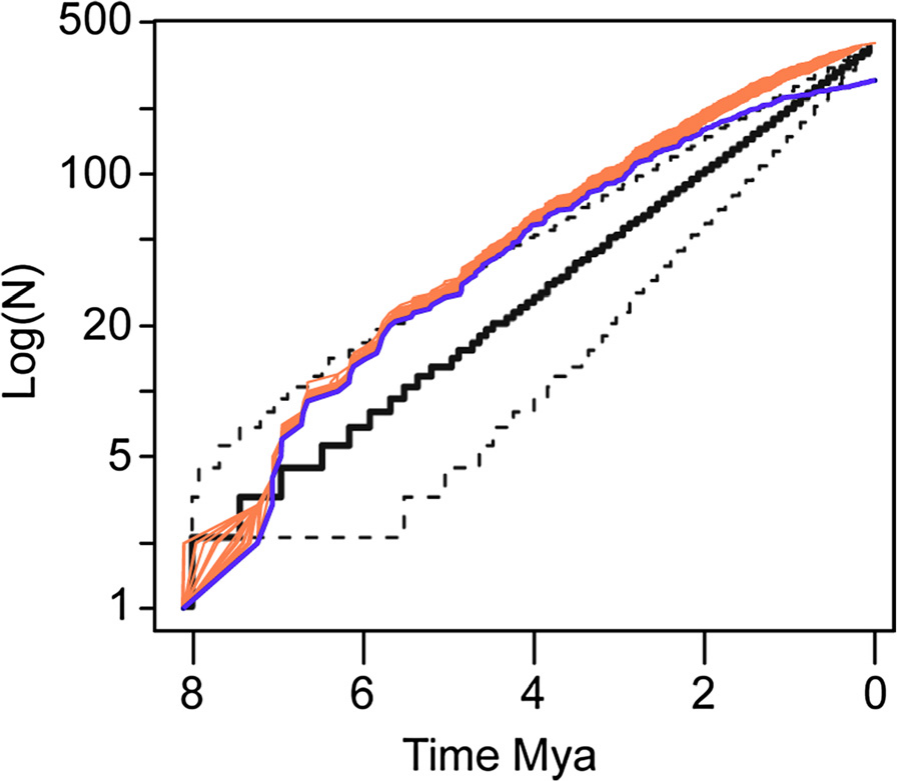
Lineage-through-time (LTT) plots. LTT plots obtained with the empirical sigmodontine phylogeny (blue line) along with the LTT plots obtained from 1000 *completed* data sets using the CorSiM approach (orange lines). Mean LTT plot (black line) and 95 % CI (dashed lines) for 1000 trees simulated under a pure-birth model.

The LTT analysis for Sigmodontinae revealed a decline in diversification toward the present (see Fig. 2). The γ-statistic indicated short internode distances clustered close to the root of the MCC tree (γ = −7.673, p < 0.01), rejecting the null hypothesis of cladogenesis under constant rates. The DDL was the best-fitting model for the MCC tree, this is a logistic density-dependent model that suggests a slowdown in diversification approaching the present (Additional file 3: Table S3).

The CorSiM method implemented to account for the bias of non-random incomplete sampling in our data estimates gave a mean γ value of −5.906 [standard deviation (SD) = 2.972.970, p < 0.05] also rejecting a constant-rate model in all of the 1000 simulations (see also the LTT plot in Fig. 2). The MCCR test rejected the hypothesis of constant rates (critical value = −3.52, p-value = 2.5 × 10^−4^). In the analysis of the *completed* dataset, Laser preferred the DDL model in 80.7.% of the cases (delta > 2 units of AIC) and DDX in the remaining fraction.WThe TreePar method selected the Yule two-rates model in 74.6 % of the simulations (delta > 2 units of AIC) and theBD2r model in the remaining cases , also indicating slowdown ~1.4 My (Table 3 and Additional file 4: Table S4).

**Table 3:** Results of fitting diversification models to the pattern of the sigmodontine radiation. Summary of diversification models fitted to the branching times derived from the the 1000 *completed* data sets generated via CorSiM (using Laser and TreePar) . Models considered: pure-birth model -PB-, birth-death -BD-, two rate-variable models [logistic density-dependent model and the exponential density-dependent model (DDL and DDX respectively)], three time-varying models [incorporating declining speciation rate, and BD2r and Y2r [variants of BD PB models with a rate shift at a certain time “st”]. Bold parameter values indicate the best fitting models according to AIC values. Parameters are abbreviated as follows: a = extinction fraction (extinction/speciation); kd = k-parameter from the DDL model; λ = speciation rate; μ = extinction rate; r = net diversification rates (speciation-extinction), r1 = initial rate and r2 = final rate; st = shift time; xp = the x-parameter from the DDX model

CorSiM (Laser)	Parameters	SD	Mean AIC	% favored model
PB	r 0.45	0.01	−2258	0
BD	r 0.45; a 0	0.01; 0	−2556	0
DDL	r 0.72; kd 635.7	0.04; 73.57	−2593	94.6
DDX	r 2.13; xp 0.3	0.12; 0.01	−2588	5.4
CorSiM (TreePar)				
PB	Λ 0.452	0	878.9	0
BD	Λ 0.452 ; μ 3.14e-7	0.0055; 7.60E-07	880.9	0
Y2r	Λ2 0.58, Λ1 0.326, st 1.405	0.033; 0.029; 0.399	850.3	88.6
BD2r	a1 16.5; a2 0.01; r1 -1.73; r2 0.61; st 1.08	71.0; 0.04; 6.99; 0.046; 0.57	852.8	11.4

**Fig. 3:**
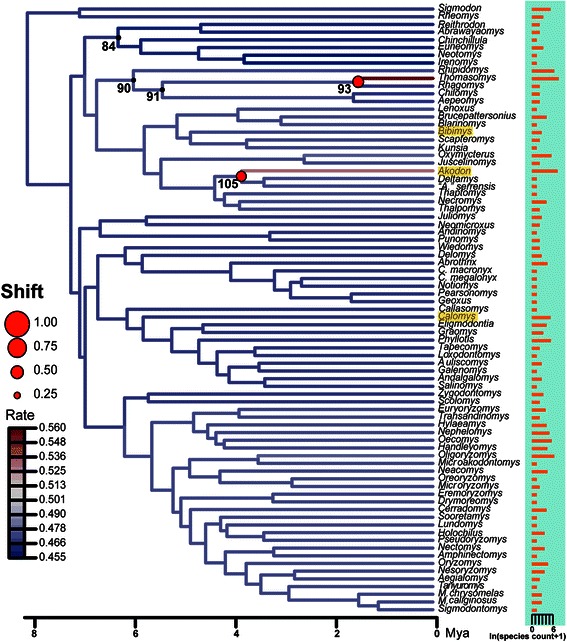
Diversification rate shifts retrieved from MEDUSA and BAMM. MEDUSA analyses were conducted using 6000 trees randomly selected from the posterior distribution found with BEAST, pruned to a backbone tree representing genera. Horizontal bars illustrate the species richness of each genus. Node numbers and circles at nodes indicate the inferred position of rate shift increases; numbers correspond to those of Additional file 5: Table S5; circles are scaled according to the proportion of 6000 sampled trees in which the shift was detected. Branches are color coded according to the magnitude of each rate shift, averaged on 6000 trees, from that of the background diversification rate. According to BAMM, the 95 % credible set of distinct shift configurations identified 4 distinct scenarios: no shift (PP = 0.86) and shifts within *Calomys* (PP = 0.042) and the branches leading to the *Akodon* (PP = 0.032) and *Bibimys* (PP = 0.031) clades. These taxa labels are highlighted in yellow.

The MEDUSA approach applied over the backbone tree favored a PB model and detected an increment in net diversification rates (stem node *Thomasomys,* Fig. 3) relative to the background sigmodontine level (log-likelihood = −278.3103, final corrected AIC = 562.7775, Table S5). When repeated through the 6000 subsampled trees, this procedure recovered 5 shifts in net diversification that were present in at least 5 % of the trees. After *Thomasomys,* the second most frequent shift was an increment in rates located in the branch leading to the *Akodon* clade (Fig. 3). Two nodes showed a decrease in net diversification: the base of Thomasomyini clade and the most recent common ancestor of Reithrodon-Irenomys (nodes 91 and 84 respectively, see also Additional file 5: Table S5).

**Fig. 4:**
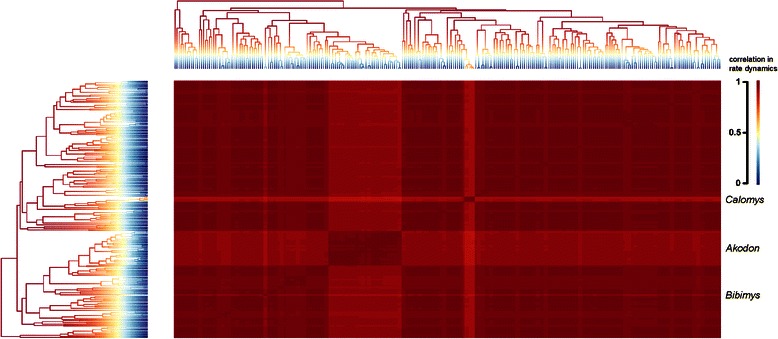
Macroevolutionary cohort matrix for the simgodontine radiation. Illustration of the macroevolutionary trends for the sigmodontine lineages which are governed by a common set of macroevolutionary rate parameters obtained with BAMM. The MCC tree is shown for reference in the left and upper margins of the figure (branches drawn with cool colors indicate slow diversification rates while warm colors indicate faster rates). The color of each individual cell indicates the average correlation in rate regimes between two species (red = identical rate). This depicts the pairwise probability of shared macroevolutionay dynamics between two lineages. Scale for the color is indicated at the right. Selected clades are identified at the right.

Among BAMM results, the overall trend in the sigmodontine radiation was summarized as a rate-through-time plot indicating a slowdown in speciation rates close to the present (Additional file 6: Figure S1). We estimated a higher mean speciation rate (0.469, 90 % HPD 0.416-0.536) than the previous estimate for Murinae (speciation - extinction = 0.42, confidence interval = 0.37-0.48, [63]). A low extinction rate (0.057, 90 % HPD 0.004-0.156) was detected for the overall phylogeny (Additional file 6: Figure S1). The PP for a model with a single evolutionary rate dynamic was 0 .71 while the PP for a model with one shift was 0.24 . There was no evidence overall for a rate shift, the BF for a scenario with one shift was 0.51. Four distinct configurations emerged after we identified a 95 % credible set of distinct shift configurations (see Fig. 3): i) no shifts (PP = 0.86), ii) a shift leading to a clade including *Calomys callosus*-*C. boliviae* (PP = 0.042), iii) a shift close to the most recent common ancestor of the *Akodon* clade (PP = 0.032), and iv) a shift close to the branch leading to the *Bibimys* clade (PP = 0.031). These results were congruent with the tree showing the relative support (BF) associated with a rate shift on each branch (Additional file 7: Figure S2). The branches close to the shifts leading to *Calomys* and *Akodon* had BF-supporting rate shifts (BF values 54.1 and 45.8 respectively). The cohort analysis did not show any major departure from the background macroevolutionary rate dynamic (Fig. 4).

### Ancestral state reconstruction and correlates of diversification

**Fig. 5:**
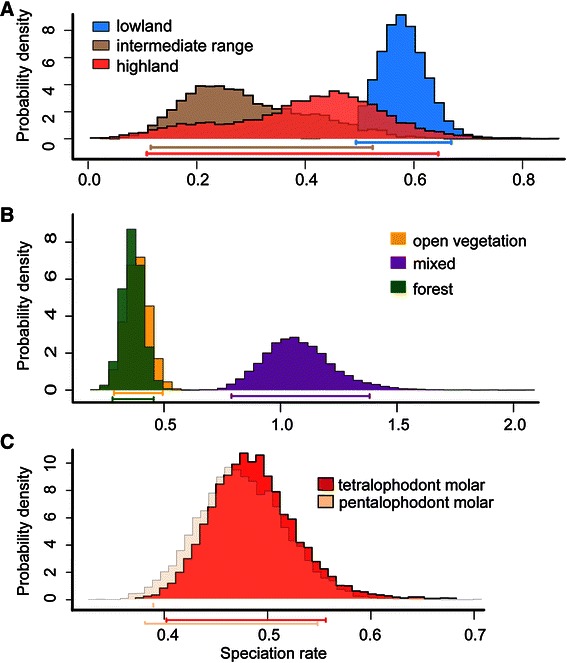
Speciation estimates inferred using MUSSE and BiSSE models. Estimates of speciation rates (λ) via MCMC chain runs for 8000 generations in each case for a full unconstrained model (see text). A) altitudinal range, B) vegetation type, and C) molar plan. The 95 % credibility intervals for each parameter are shaded and indicated by bars along the x axis.

The stochastic mapping of lifestyle or habitat type preference (elevational range and vegetation type) and the transitions between molar types using the MCC tree are shown in Additional file 8: Figure S3, Additional file 9: Figure S4, Additional file 10: Figure S5. The fit and parameters of MuSSE and BiSSE models evaluating the mode of evolution and diversification rates for the transition across altitudinal range, vegetation type and molar architecture across the MCC tree are summarized in Additional file 11: Table S6 and Fig. 5. Comparisons of models using AIC provided the best fit when separate rates of speciation in different altitudinal ranges were allowed. Under a time-dependent model, following a shift inferred at 3.16 Mya, there was a slowdown in speciation rate at the highlands and an increase in speciation rate in the mid range towards the present. Likewise, for the full model, the lowlands sustained higher speciation rates than the highlands. For the association between vegetation type and speciation rates, the time-dependent model was the one that best fitted the data according to AIC . This model suggested a slowdown in speciation rates in open and forest habitats, but no abrupt change was observed for habitat containing mixed vegetation. In the epoch close to the present -as well as in the full model- lineages inhabiting mixed vegetation exhibited higher speciation rates than those present in the remaining habitats. Considering the 2-epochs model, the lineages occupying highlands exhibited a decrease in extinction rates towards the present. While in mixed vegetation we inferred an increase in extinction rates there was an decrease in extinction rates in lineages inhabiting the forest. The BiSSE models found no support for different speciation rates associated with a particular molar type when comparing full and constrained models, although the time-dependent model performed better in AIC scores. Bayesian posterior distribution for these trait-dependent speciation rates estimated under for the full models are shown in Fig. 5.

Considering the full models, the greater transition rates between vegetation types were found from mixed type into the open vegetation. In the case of the altitudinal range the greater transition rates were observed from middle range into the lowlands and from the highlands into middle ranges. Transitions in molar types were more frequent from the tetralophodont to the pentalophodont condition; the latter morph is inferred as the derived state (see Discussion, Additional file 12: Table S7).

### Geographic range evolution

**Fig. 6:**
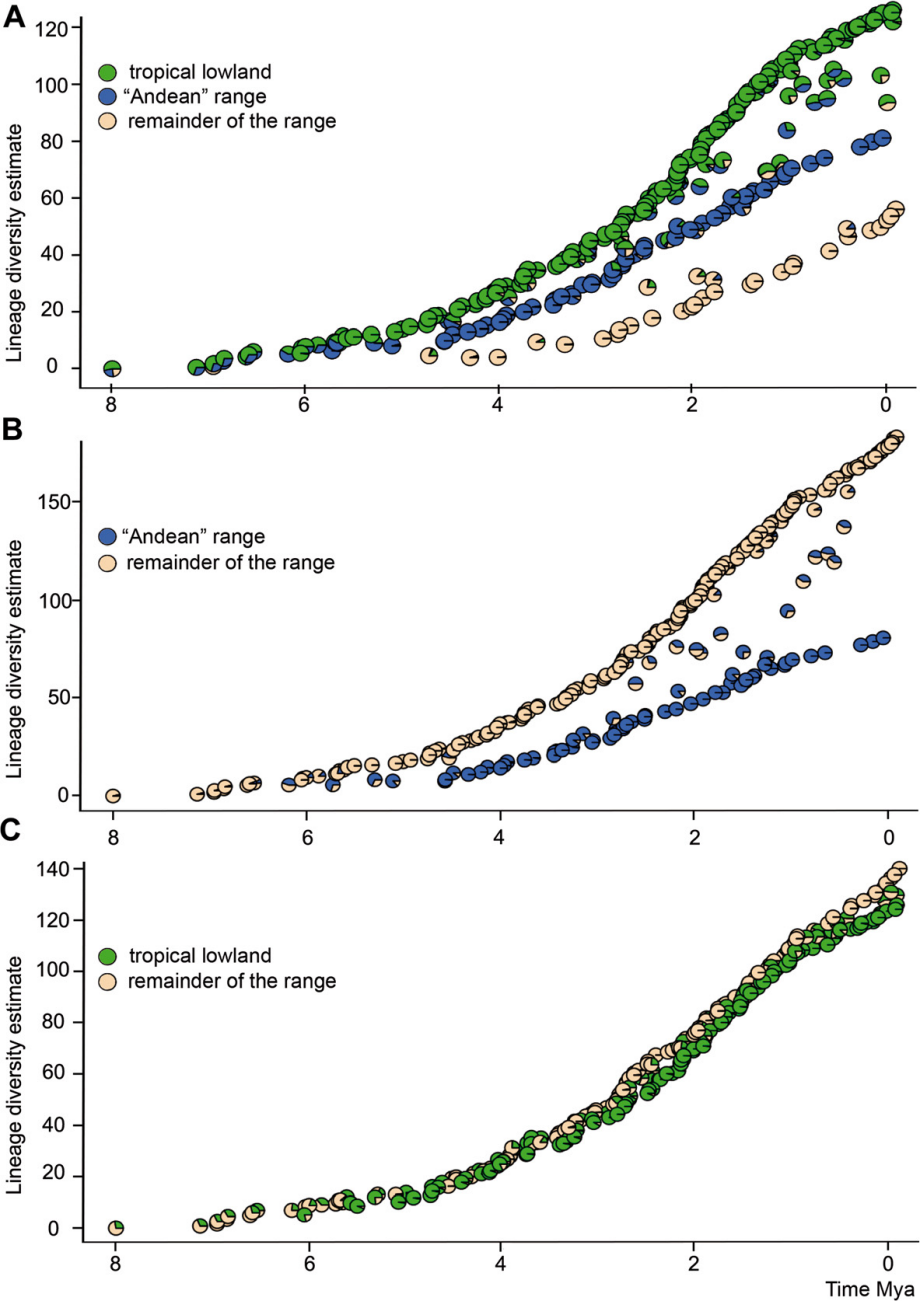
Sigmodontine species accumulation estimates over time. Comparison of historical lineage diversity estimates and relative branching time estimates for each node in the MCC tree. Pie diagrams are color coded according to ancestral state reconstruction of occurrence in distinct areas. (**a**) comparison between a composite area of Andean, Paramo, Patagonian, and Puna biomes, an area including Amazonas, Cerrado, Caatinga, Parana, and Yungas; (**b**) occurrence in “Andean” range versus remainder of the distribution; (**c**) occurrence in tropical lowlands versus remainder of the distribution.

In our sets of models, those with distinct speciation rates between geographic areas were preferred over models with a single speciation rate or for those with identical cladogenesis within a single region (Additional file 12: Table S7). A model constrained with equal in situ speciation rate (λ_111_ and λ_222_ in the model) was favored in only one in four of the model sets (i.e. when the Yungas province was included as part of the Andean area). In summary, these analyses recovered higher speciation rates within the tropical lowlands than within the remainder distributional area of the group. When plotted the accumulation of lineages within our distinct regions of interest, it revealed that lineage diversity has been higher across the area defined as “tropical lowlands” during the last ~4 Mya (Fig. 6).

## Discussion

### Divergence time estimates

Our analyses, employing a dense taxon sampling, provided a framework for examining the influence of habitat and geographical context on the diversification of a large group of Neotropical rodents. Given that the phylogenies obtained are topologically in agreement with those recovered in previous studies (e.g., strong support for the monophyly of Sigmodontinae, Oryzomyialia, and all tribes; lack of resolution for relationships among tribes within Oryzomyalia; Table 1; see also [10, 20–26, 61, 62]), here we will focus on the general pattern of diversification. Our estimates of the crown age of Sigmodontinae and its main clades, most of which are ranked as tribes, are consistent with the ones found previously (Table 2 and references therein). The divergence estimates are younger than those previously found (although with overlapping confidence intervals) probably due to the use of a different calibration scheme.

### Diversification patterns

We employed several strategies, including a data augmentation procedure which simulates missing species to generate “complete” data sets, to examine the diversification patterns in sigmodontine rodents. Considering both the MCC tree and the *completed* data sets, from the analysis of γ-statistic and LTT plots and a model-fitting approach, we detected a lineage-wide slowdown in the sigmodontine diversification since the beginning of the Pleistocene. The rates-through-time analysis for the MCC tree in BAMM also supported this finding. In addition, we found two lines of evidence suggesting among-lineage variation in diversification rates during the radiation of the subfamily. Firstly, the MEDUSA analysis detected shifts at the nodes leading to *Akodon* and *Thomasomys*, which are both species-rich clades. Additionally the decrease in net diversification at two nodes might suggest a decline in birth rates -or an increase in extinction rates- in certain phases within the radiation. Although the BAMM method did not provided strong overall evidence for a rate shift, three clades within the tribes Akodontini (*Akodon*, *Bibimys*) and Phyllotini (*Calomys*) exhibited rate shifts according to our recovered 95 % credible set of shifts although these represent ~10 % of PP. It must be noted that, to account for incomplete sampling, MEDUSA employs a backbone tree while BAMM, in addition to relaxing the assumption of time-homogeneous diversification used in most previous approaches, operates over the empirical phylogeny without any pruning [48]. BAMM also accounts for non-random incomplete sampling via its per-clade sampling probabilities. These differences might explain the discrepancies among results of both analyses; although both of them inferred a change in regimes close to the *Akodon* clade. A recent study [21] found shifts in the branches leading to the clades of *Akodon* and *Oxymycterus*. On the other hand, Schenk et al. [26] detected rapid shift at the base of Oryzomalia, close to the base of Oryzomyini and at a node leading to Abrotrichini-Phyllotini-Akodontini. The discrepancies between our results and those from other studies [21, 25, 26] might be due to the differences in the taxonomic sampling and the branching pattern recovered at the base of Oryzomyalia.

Our results are in line with recent studies that also found diversification slowdowns for other Neotropical radiations (e.g., [60, 64] although there is much discussion on how to interpret this pattern (e.g., [65–67]. In our case it is hard to point a single biological explanation that might underlie the observed diversification slowdowns and the shifts in rates observed in some lineages. Diversification slowdowns, usually linked to diversity-dependent speciation are also associated with competition for limited resources or ecological limits on the number of species in a clade (e.g., [46, 68]) but alternative explanations exist. As lineages accumulate, it is expected that species ranges become more subdivided as speciation promoted by vicariance diminishes [68]. If range expansion is inhibited, the geographic context can lead to diversification slowdowns without niche differentiation [46, 69]. Another explanation invokes the failure to include incipient species as distinct units in our data, excluding in such way from the analyses recent cladogenetic events [70, 71]. This issue probably affects our dataset as taxonomists are less likely to differentiate, and then identify, newly formed species than those that have accumulated more changes after having originated earlier. If this occurs, the reconstructed phylogeny would exhibit a decline in the diversification close to the present [46]. In addition to this effect (which could affect the entire reconstructed tree), we note that the taxonomic effort given to explore sigmodontine diversity differs in intensity and approaches among genera and tribes and also geographic regions (see comments in [8]). There are poorly studied groups and others that have not yet been reviewed with a more integrative contemporaneous approach. Furthermore, it is arguable whether species diversity is better known in the open areas of central and southern South America, than in Amazonia and the tropical Andes [8], but see [72, 73] for example on Andean endemism of mammals -especially rodents- at middle to upper elevations. This might partially explain why some groups like *Akodon*, which is relatively well known, exhibited branch-specific shifts in diversification rate. Also chromosomal variation exhibited by *Akodon* [74]may have influenced species designationsin this taxon. However, we note that the taxonomy of the diverse *Thomasomys*, for which we also infer a branch-specific shift in diversification rates using MEDUSA, is mostly based on morphologic evidence [75].

The biogeographic scenario where *Calomys* and *Akodon* proliferated seems to have been crucial for the radiation of these genera since both taxa present a similar distributional range through open and forested areas both north and south of Amazonia and along the Andes, extending south into Patagonia. *Thomasomys*, which also showed rate shifts, has in turn proliferated through the Andean highlands and forested areas. It must be noted that no rate shifts were inferred for other species-rich genera like *Oligorzymoys* or *Phyllotis* where the accumulation of diversity maintained a steady rate since the Pliocene. In order to discriminate between the predictions from niche differentiation and alternative hypotheses, more data, including species range overlap, divergence along the ecological axis as well as robust and resolved within genus phylogenies, are needed.

In contrast to the pervasive species richness of some clades, our topologies corroborated the existence of several deep sigmodontine lineages constituted by a single living genus composed by only one, two or three species (e.g., those leading to *Delomys*, *Juliomys*) and that are not associated to any fossil genus [11]. These depauperate lineages contrast with other lineages of similar phylogenetic deep that proliferated extensively (e.g., Abrotrichini, Akodontini or Oryzomyini). Historical contingency or local determinism might explain why some lineages apparently “failed” to radiate. According to some authors (e.g., [26, 76]) after early colonizers take over existing resources, late arrivals might not radiate. Also due to lack of speciation, species accumulation might also decrease even when resources are available [77].

### Correlates of diversification

Our results suggest that vegetation type and altitudinal range are correlated with sigmodontine diversification rates. Clades that occupied mixed vegetation areas presented the highest diversification rates, which is in agreement with what was suggested by Smith et al. [78]. Likewise, lowland lineages supported higher diversification rates than those occurring in highlands. It must be noted that the categorical definition of low and highlands used here is, at best, a proxy for other factors, such as temperature, spatial heterogeneity or humidity, all of which might affect the life history of organisms and species attributes (e.g., distributional range). Together with other potential influential factors (i.e., the ongoing Andean orogeny; see review in [79]), the advance into new areas during the Late Miocene and the access to new resources found in open vegetation or lowland habitats might have prompted higher rates of speciation. Additional data are needed to corroborate this scenario and to identify the processes (e.g., ecological release [80]) behind this pattern.

We also examined the hypothesis linking transitions in molar morphology with opportunity to diversify within Sigmodontinae. In classic studies (e.g., [18, 81, 82]), the pentalophodont condition is regarded as the sigmodontine plesiomophic state (Additional file 10: Figure S5). Our results indicate that the pentalophodont condition is derived; however, this result is somewhat questionable because support is lacking for relationships at the more basal nodes of the Oryzomyalia. More importantly, independent of which molar type is ancestral or derived, results suggest that the acquisition or loss of a fifth molar loph was not a key innovation in sigmodontine evolution since we found no correlation between the evolution of this trait and speciation rates. However, we cannot exclude the possibility that molar morphology is associated or covaries with other traits that influenced the sigmodontine radiation. Finally, we are aware of methodological drawbacks in using BiSSE models when the taxonomic coverage is not exhaustive or below 300 terminals ([83], see [84] for a discussion on model inadequacy), so further examination of these trends with other methods is necessary.

### Andean region and tropical lowlands as promoters of diversity

During the Late Miocene, sigmodontine rodents dispersed across newly emerged habitats finding ample opportunities to radiate in South America. The Andean region, or more broadly speaking the South America western highlands, played an important role in the diversification of the Neotropical biota [3, 16, 85]. Along this line, Reig [9] proposed the Andes were the “areas of original differentition” for most sigmodontine tribes. Our data best fit a model accounting for higher speciation rates and species accumulation within the “tropical lowlands” (a wide area composed of Amazonia and the eastern Cerrado, Caatinga and Parana regions and also the Yungas) than in the Andean region. Several issues might explain the incongruence between our results and the “traditional” view emphasizing the role of Andean regions in the proliferation of these rodents. Field work in the last two decades have revealed far more sigmodontine phylogenetic richness outside the Andes than formerly documented (e.g., [86][61]; but see also [87]). Moreover, phylogenetic analyses (e.g., [23] ) have prompted distinct delimitations (in some cases radically distinct) of the main sigmodontine groups (i.e., tribes) than those envisioned earlier. Our results suggest that Andean regions might have acted as a “species pump” [88, 89] pouring taxa that in turn radiated in the newly emerged habitats [90] and also acting as the receptor of main lineages originated elsewhere. In turn, the tropical lowlands appeared to have acted both as a cradle for new species (sustaining high speciation rates) and also as a museum that preserved old lineages that began to radiate outside this region (see also [89]). Further work would improve our understanding of the polarity and timing of transitions between these areas.

### Methodological limitations

Inferring diversification patterns from large time-calibrated phylogenies has proven to be a complex task [42, 67, 91]. Incomplete taxon sampling can pose a major problem to such analyses [92–94]. We addressed some common issues that could limit our analysis in two ways. While applying the MEDUSA method, we examined several trees from the posterior distribution to account for the uncertainty of phylogenetic reconstruction. We also considered new approaches that account for missing lineages [CorSiM and BAMM [43, 47], thereby improving hypothesis testing frameworks. It must be noted that the MuSSE, BiSSE and ClaSSE results depend on an empirical MCC tree and do not account for non-random incomplete sampling. Another relevant limitation of our study is that our phylogenetic reconstructions are based on sequences of two loci and that for some taxa only one gene was available; as such nodes were weakly supported. Notwithstanding, some methodological limitations might hamper the interpretation of our results but future work should clarify or contextualize some issues (e.g., the relevance of the geographic range).

## Conclusions

This study provided a new chronology for the evolution of the subfamily Sigmodontinae under the most taxon-dense sampling up to date. In particular, here we focus on the main pattern and tempo of the sigmodontine radiation rather than centering in particular clades. We present evidence indicating that the evolution of habitat preference or transitions between vegetation type and elevational range were associated with diversification rates. Our results also highlight the importance of the tropical lowlands, an area with the highest accumulation of species through time. In order to assess the ecological constraints tied to this group, proxies for niche space, lifestyle traits, biologically important phenotypic features such as body size or species' geographic range are needed. We note that information for some traits of interest is currently unavailable for important fractions of the sigmodontine radiation, but in the future more data should be available. Even with the aforementioned limitations, our analyses have proven to be useful to understand the emerging pattern of diversification in the Neotropics (e.g., [60, 72, 89, 95] providing hypotheses aimed to understand this diverse and ubiquitous group of Neotropical mammals.

## Availability of supporting data

The data set supporting the results of this article is available in the LabArchives repository, available here http://dx.doi.org/10.6070/H42J68TX.

## Additional files

Additional file 1: Table S1. Detail of specimens examined in this study. **GenBank accession numbers**, trait values, and distributional range for the species included in this study. Traits were coded as detailed in the “Material and Methods” section. Vegetation: 1) open vegetation, 2) mixed, or 3) forest. Altitudinal range: 1) lowlands, 2) middle range, or 3) highland. Molar type was coded as 0) tetralophodont, or 1) pentalophodont. The distribution coded for the ClaSSE models is listed under the “Area” column 1) remainder of the distribution, 2) Andean, and 3) tropical lowlands. All GenBank accession numbers for analyzed sequences are given in the last two columns. “N” indicates missing data. (XLS 44 kb) Additional file 2: Table S2. Taxonomic richness paired along the backbone tree employed in the MEDUSA analysis. (XLS 11 kb) Additional file 3: Table S3. Results of fitting diversification models to the empirical phylogeny of Sigmodontinae. Summary of diversification models fitted to the branching times derived from the empirical phylogeny of Sigmodontinae (MCC tree). Models considered: pure-birth model -PB-, birth-death -BD-, two rate-variable models [logistic density-dependent model and the exponential density-dependent model (DDL and DDX respectively)], three time-varying models [incorporating declining speciation rate , increasing extinction and a model where both rates can vary (SPVAR, EXVAR, BOTH respectively)], and BD2r and Y2r [variants of BD PB models with a rate shift at a certain time “st”]. Bold parameter values indicate the best fitting models according to AIC values. Parameters are abbreviated as follows: a = extinction fraction (extinction/speciation); k = parameter exponential change in speciation rate; kd = k-parameter from the DDL model; λ = speciation rate; μ = extinction rate; r = net diversification rates (speciation-extinction), r1 = initial rate and r2 = final rate; st = shift time; xp = the x-parameter from the DDX model and z = parameter exponential change in extinction rate. ^a^ time-varying model (XLS 7 kb) Additional file 4: Table S4. Results of fitting diversification models to the *completed* data sets for the sigmodontine radiation in Laser and TreePar. Table showing the diversification models fitted to the branching times derived from the 1000 *completed* data sets generated via CorSiM. For each simulation (#sim) AIC values and Delta AIC are given. AIC values for the best fitting models according AIC are highlighted in bold numbers. Models considered: pure-birth model -PB-, birth-death -BD-, two rate-variable models [logistic density-dependent model and the exponential density-dependent model (DDL and DDX respectively)] and BD2r and Y2r [variants of BD PB models with a rate shift at a certain time “st”]. In the Laser analysis, parameters are abbreviated as follows: λ = speciation rate; μ = extinction rate; r = net diversification rates (speciation-extinction), kd = k-parameter from the DDL model; xp = the x-parameter from the DDX model. In the TreePar analysis: r1 = initial rate and r2 = final rate; a = extinction fraction (extinction/speciation); st = shift time. Mean and standard deviation of AIC, delta AIC and parameters values are given for the entire data set. (XLS 775 kb) Additional file 5: Table S5. Results of the MEDUSA analysis for the sigmodontine radiation. MEDUSA analysis were conducted using 6000 trees randomly selected from the posterior distribution found in BEAST, pruned to a backbone tree representing only genera. Node numbers correspond to those in Fig. 3. Shifts are indicated as magnitudes of difference over the background rate estimated from the data. (XLS 6 kb) Additional file 6: Figure S1. Rates-through-time trajectories for the sigmodontine phylogeny obtained with BAMM. Speciation-through-time shown in red and extinction-through-time shown in blue. Shaded polygon denotes the 5 % through 95 % Bayesian credible regions on the distribution of rates at any point in time. (TIFF 137 kb) Additional file 7: Figure S2. Phylogeny of sigmodontine rodents including the probability of rate shifts. Following the BAMM analysis, branch lengths were drawn proportional to their Bayes factor evidence for a rate shift. Bayes factors greater than 5 are highlighted in red. (TIFF 513 kb) Additional file 8: Figure S3. Ancestral state reconstruction for the altitudinal range of sigmodontine rodents. Altitudinal ranges are illustrated by colored bars on the tips of the branches. Pies at internal nodes represent ancestral probabilities of the traits recovered by simmap. (TIFF 866 kb) Additional file 9: Figure S4. Ancestral state reconstruction for the vegetation type occupied for sigmodontine rodents. Habitats associated with vegetation are illustrated by colored bars on the tips of the branches. Pies at internal nodes represent ancestral probabilities of the traits recovered by simmap. (TIFF 766 kb) Additional file 10: Figure S5. Ancestral state reconstruction for the molar plan of sigmodontine rodents. Molar morphology is illustrated by colored bars on the tips of the branches. Pies at internal nodes represent ancestral probabilities of the traits recovered by simmap. (TIFF 725 kb) Additional file 11: Table S6. Comparison of full and constrained maximum Multiple State Speciation and Extinction (MuSSE) models considering altitude and vegetation and Binary State Speciation and Extinction (BiSSE) models for the molar plan. Altitudinal range was treated as follows: 1) lowland; 2) middle range; 3) highland. Vegetation type was treated as follows: 1) open-vegetation; 2) mixed; 3) forest. Molar architecture was treated as follows: 0) tetralophodont, or 1) pentalophodont. λ = trait specific speciation rates; μ = traits specific extinction rates; q = transition rate parameters. Constraints for the MuSSE models are as follows: 1) all λ equal, all μ equal, q_21_ ~ _q12_ ~ q_13_ ~ q_32_ ~ q_23_, 2) all μ equal, q_21_ ~ _q12_ ~ q_13_ ~ q_32_ ~ q_23_, 3) all λ equal. The 2-epochs model considers different parameters established by a point in the past that was previously estimated in a likelihood framework. *Epoch 1* spans from the present to 3.16 Mya (for the altitude model), 2.77 Mya (in the case of vegetation and molar models), while *Epoch 2* extends to the root. Models are compared using the Akaike Information Criterion (AIC); log-likelihood (lnLik) and delta AIC values (Delta) are given in the table. The model with the lowest AIC score is shown in bold. (XLS 12 kb) Additional file 12: Table S7. Association between species range and diversification. Comparison of full and constrained maximum Cladogenetic State change Speciation and Extinction (ClaSSE) models evaluating the geographic range of members of Sigmodontinae. For the “Andean” model geographic range was coded as: 1) present in an area composed of Andean, Paramo, Puna, and Patagonian regions, or 2) present in the remainder of the distribution of Sigmodontinae. For the “tropical lowlands” model geographic range was treated as: 1) present in an area composed of Amazonian, Cerrado-Caatinga, and Parana regions, or 2) present in the remainder of the distribution of Sigmodontinae. λ = range specific speciation rates; μ = range specific extinction rate; q = transition rate parameters. Constraints were as follows: 1) μ_1_ ~ μ_2_, q_12_ ~ q_21_, 2) μ_1_ ~ μ_2_, 3) q_12_ ~ q_21_, 4) all λ rates equal, μ_1_ ~ μ _2_, q_12_ ~ q_21_, 5) 2 rates λ_222_ ~ λ_111_, λ_122_ ~ λ_112_ ~ λ_211_ ~ λ_212_, μ_1_ ~ μ_2_, q_12_ ~ q_21;_ 6) 3 rates: λ_222_, λ_111_, λ_122_ ~ λ_112_ ~ λ_211_ ~ λ_212_, μ_1_ ~ μ _2_, q_12_ ~ q_21;_ 7) 4 rates λ_222_, λ_111_, λ_212_ and all remaining rates are equal, μ_1_ ~ μ _2_, q_12_ ~ q_21_. Models are compared using the Akaike Information Criterion (AIC), delta values for the AIC and log-likelihood (LnLik) values are shown. The models with the lowest AIC scores are in bold. The first set of models have the Yungas included as part of the “tropical lowland” distribution while in the alternative models set the Yungas are considered as part of the “Andean” distribution. (XLS 15 kb)

## Abbreviations

AIC: Akaike Information Criterion
BD: Birth-Death
BD2r: a variant of birth-death model with a rate shift at a certain time “st”
BF: Bayes Factor
BiSSE: Binary State Speciation and Extinction model BOTHVAR: model with variable speciation and extinction
ClaSSE: Cladogenetic State change Speciation and Extinction Model
Cyt b: Cytochrome b
DDL: Density-dependent model of speciation model, logistic variant
DDX: Density-dependent model of speciation model, exponential variant
ESS: Effective sample size
EXVAR: Model with exponentially increasing extinction and constant speciation
HPD: Highest posterior density
IRBP: Interphotoreceptor retinoid-binding protein
LnLik: log likelihood
LTT: Lineages through time
MCC: Maximum clade credibility
MuSSE: Multiple State Speciation and Extinction model Mya, Million years ago
PB: Pure Birth model of diversification
PP: Posterior Probability
SD: Standard deviation
SPVAR: Model with an exponentially declining speciation rate through time and constant extinction
st: Time when a model has a rate shift
TVM: Transversional nucleotide substitution model
Y2r: Variant of PB model with a rate shift at a certain time “st”
