# Supplementary figures and images for: The influence of ecological and geographical context in the radiation of Neotropical sigmodontine rodents
Source: BMC Evol Biol. 2015 Aug 26;15:172. doi: 10.1186/s12862-015-0440-z (PMC4549906; doi:10.1186/s12862-015-0440-z)

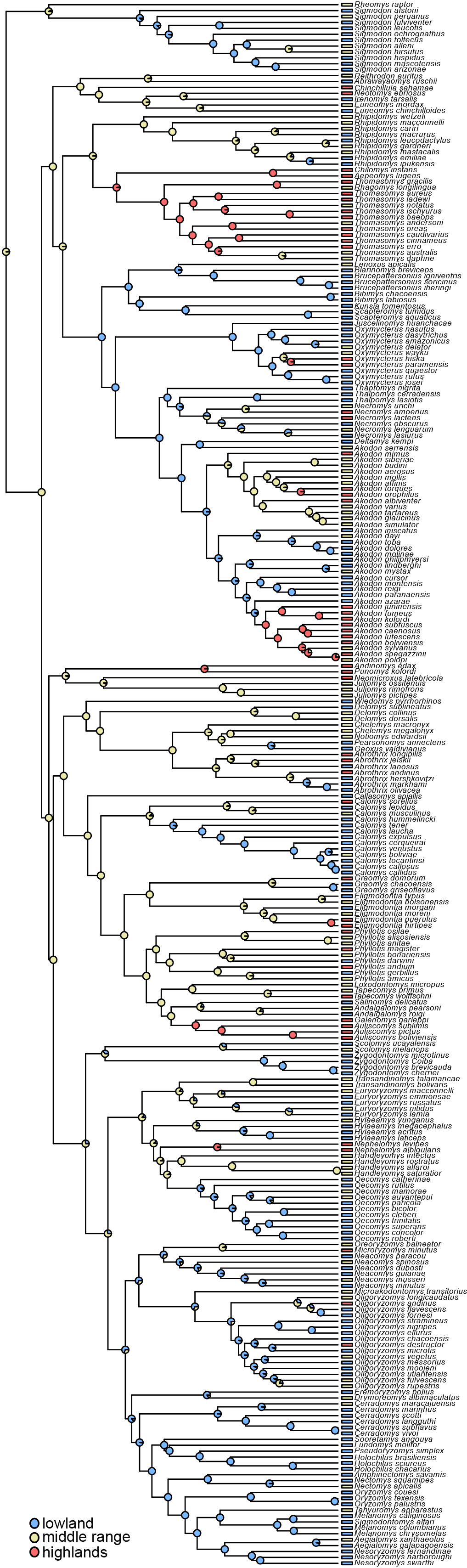

Supplement: Additional file 6: Figure S1. — Rates-through-time trajectories for the sigmodontine phylogeny obtained with BAMM. Speciation-through-time shown in red and extinction-through-time shown in blue. Shaded polygon denotes the 5 % through 95 % Bayesian credible regions on the distribution of rates at any point in time. (TIFF 137 kb) [file 12862_2015_440_MOESM8_ESM.tif]

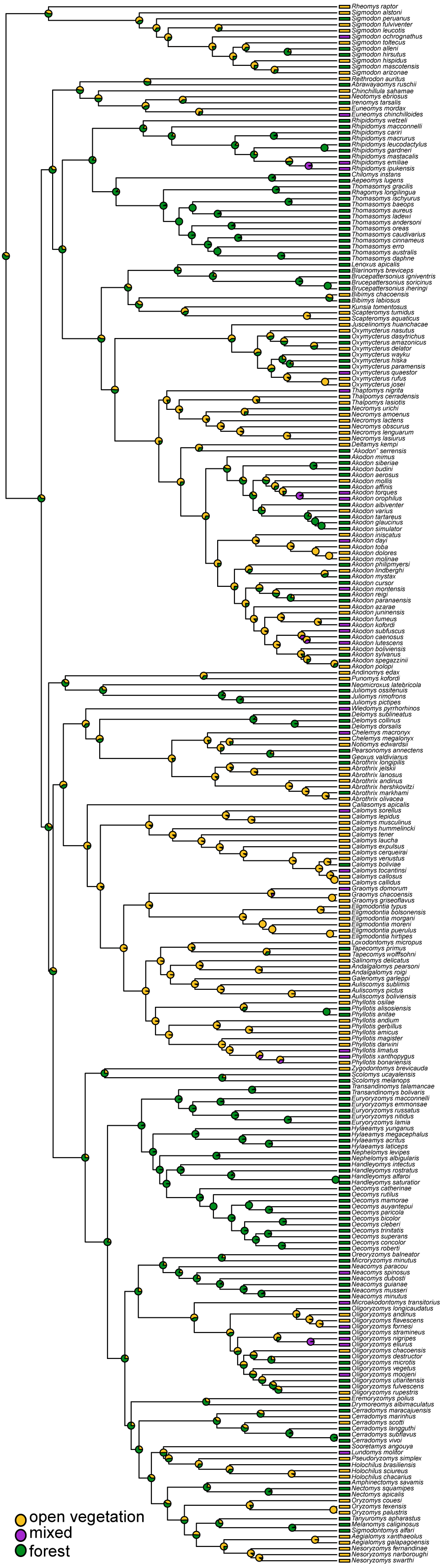

Supplement: Additional file 7: Figure S2. — Phylogeny of sigmodontine rodents including the probability of rate shifts. Following the BAMM analysis, branch lengths were drawn proportional to their Bayes factor evidence for a rate shift. Bayes factors greater than 5 are highlighted in red. (TIFF 513 kb) [file 12862_2015_440_MOESM9_ESM.tif]

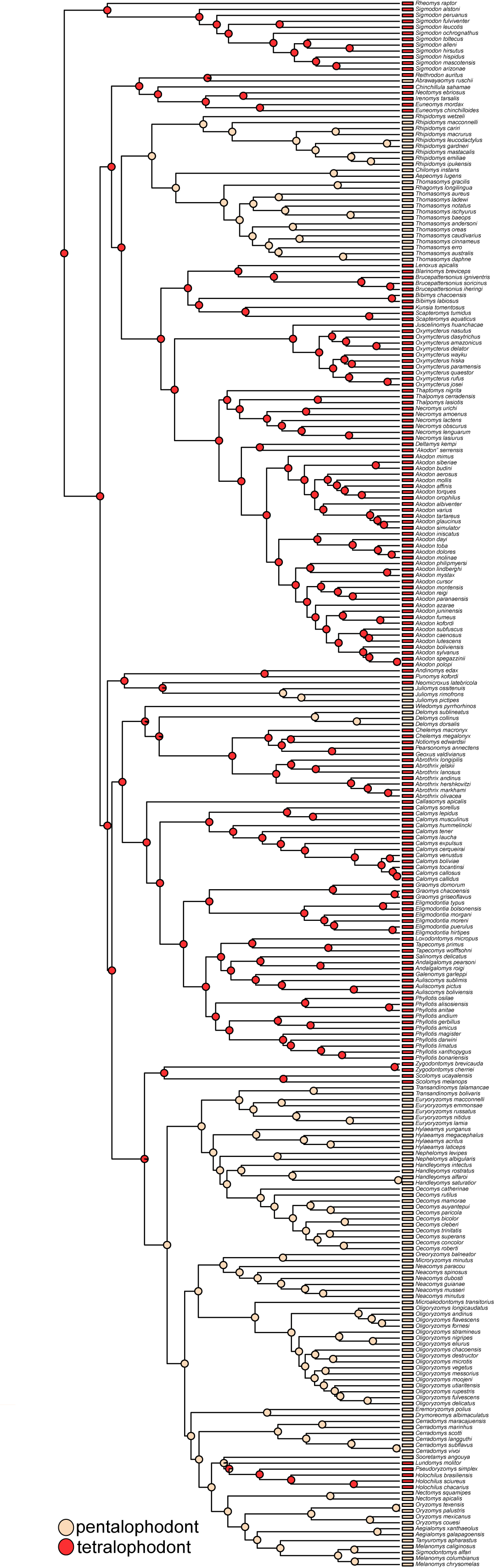

Supplement: Additional file 8: Figure S3. — Ancestral state reconstruction for the altitudinal range of sigmodontine rodents. Altitudinal ranges are illustrated by colored bars on the tips of the branches. Pies at internal nodes represent ancestral probabilities of the traits recovered by simmap. (TIFF 866 kb) [file 12862_2015_440_MOESM10_ESM.tif]

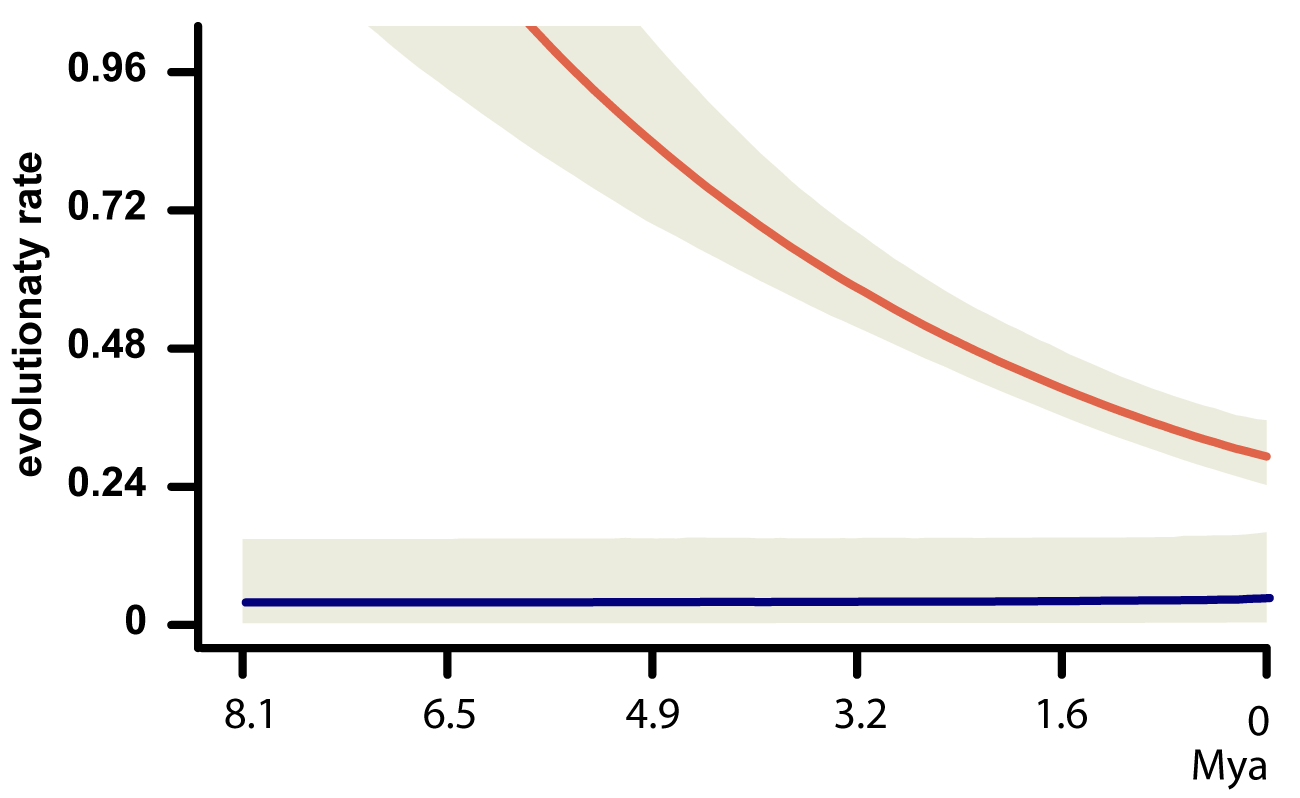

Supplement: Additional file 11: Table S6. — Comparison of full and constrained maximum Multiple State Speciation and Extinction (MuSSE) models considering altitude and vegetation and Binary State Speciation and Extinction (BiSSE) models for the molar plan. Altitudinal range was treated as follows: 1) lowland; 2) middle range; 3) highland. Vegetation type was treated as follows: 1) open-vegetation; 2) mixed; 3) forest. Molar architecture was treated as follows: 0) tetralophodont, or 1) pentalophodont. λ = trait specific speciation rates; μ = traits specific extinction rates; q = transition rate parameters. Constraints for the MuSSE models are as follows: 1) all λ equal, all μ equal, q21 ~ q12 ~ q13 ~ q32 ~ q23, 2) all μ equal, q21 ~ q12 ~ q13 ~ q32 ~ q23, 3) all λ equal. The 2-epochs model considers different parameters established by a point in the past that was previously estimated in a likelihood framework. Epoch 1 spans from the present to 3.16 Mya (for the altitude model), 2.77 Mya (in the case of vegetation and molar models), while Epoch 2 extends to the root. Models are compared using the Akaike Information Criterion (AIC); log-likelihood (lnLik) and delta AIC values (Delta) are given in the table. The model with the lowest AIC score is shown in bold. (XLS 12 kb) [file 12862_2015_440_MOESM6_ESM.tif]

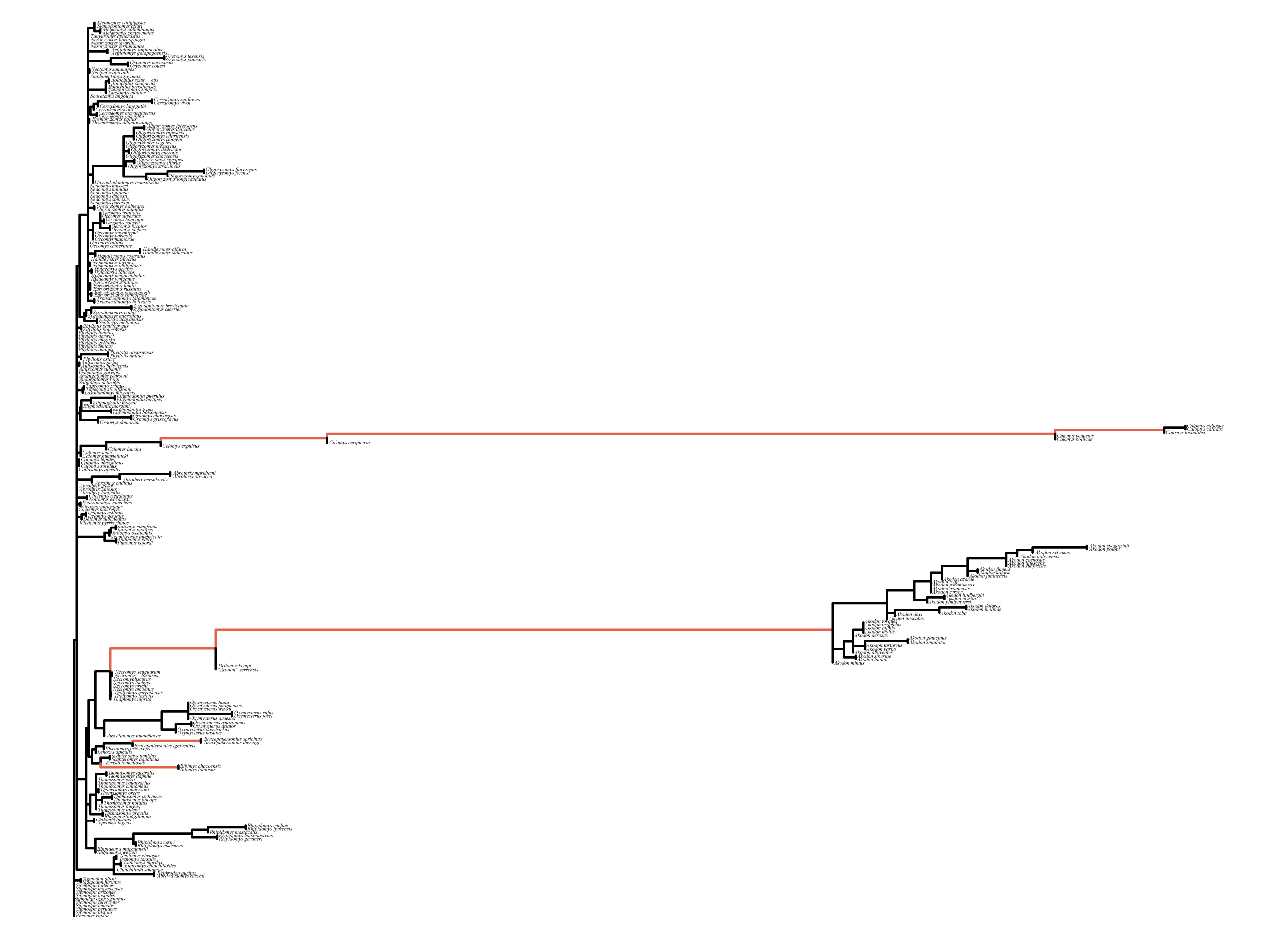

Supplement: Additional file 12: Table S7. — Association between species range and diversification. Comparison of full and constrained maximum Cladogenetic State change Speciation and Extinction (ClaSSE) models evaluating the geographic range of members of Sigmodontinae. For the “Andean” model geographic range was coded as: 1) present in an area composed of Andean, Paramo, Puna, and Patagonian regions, or 2) present in the remainder of the distribution of Sigmodontinae. For the “tropical lowlands” model geographic range was treated as: 1) present in an area composed of Amazonian, Cerrado-Caatinga, and Parana regions, or 2) present in the remainder of the distribution of Sigmodontinae. λ = range specific speciation rates; μ = range specific extinction rate; q = transition rate parameters. Constraints were as follows: 1) μ1 ~ μ2, q12 ~ q21, 2) μ1 ~ μ2, 3) q12 ~ q21, 4) all λ rates equal, μ1 ~ μ 2, q12 ~ q21, 5) 2 rates λ222 ~ λ111, λ122 ~ λ112 ~ λ211 ~ λ212, μ1 ~ μ2, q12 ~ q21; 6) 3 rates: λ222, λ111, λ122 ~ λ112 ~ λ211 ~ λ212, μ1 ~ μ 2, q12 ~ q21; 7) 4 rates λ222, λ111, λ212 and all remaining rates are equal, μ1 ~ μ 2, q12 ~ q21. Models are compared using the Akaike Information Criterion (AIC), delta values for the AIC and log-likelihood (LnLik) values are shown. The models with the lowest AIC scores are in bold. The first set of models have the Yungas included as part of the “tropical lowland” distribution while in the alternative models set the Yungas are considered as part of the “Andean” distribution. (XLS 15 kb) [file 12862_2015_440_MOESM7_ESM.tif]
